# *Organ-on-a-chip technology:* a novel approach to investigate cardiovascular diseases

**DOI:** 10.1093/cvr/cvab088

**Published:** 2021-03-17

**Authors:** Valentina Paloschi, Maria Sabater-Lleal, Heleen Middelkamp, Aisen Vivas, Sofia Johansson, Andries van der Meer, Maria Tenje, Lars Maegdefessel

**Affiliations:** 1 Department for Vascular and Endovascular Surgery, Technical University Munich, Klinikum Rechts der Isar, Munich, Germany; 2 German Center for Cardiovascular Research (DZHK), Partner Site Munich Heart Alliance, Berlin, Germany; 3 Research Institute of Hospital de la Santa Creu i Sant Pau, IIB Sant Pau, Genomics of Complex Diseases Group, Barcelona, Spain; 4 Cardiovascular Medicine Unit, Department of Medicine, Karolinska Institutet, Stockholm, Sweden; 5 BIOS/Lab on a Chip, University of Twente, Enschede, The Netherlands; 6 Applied Stem Cell Technologies, University of Twente, Enschede, The Netherlands; 7 Department of Materials Science and Engineering, Science for Life Laboratory, Uppsala University, Uppsala, Sweden; 8 Molecular Vascular Medicine Unit, Department of Medicine, Karolinska Institutet, Stockholm, Sweden

**Keywords:** Organs-on-chips, Cell culture, Cardiovascular, Heart, Personalized medicine

## Abstract

The development of organs-on-chip (OoC) has revolutionized *in vitro* cell-culture experiments by allowing a better mimicry of human physiology and pathophysiology that has consequently led researchers to gain more meaningful insights into disease mechanisms. Several models of hearts-on-chips and vessels-on-chips have been demonstrated to recapitulate fundamental aspects of the human cardiovascular system in the recent past. These 2D and 3D systems include synchronized beating cardiomyocytes in hearts-on-chips and vessels-on-chips with layer-based structures and the inclusion of physiological and pathological shear stress conditions. The opportunities to discover novel targets and to perform drug testing with chip-based platforms have substantially enhanced, thanks to the utilization of patient-derived cells and precise control of their microenvironment. These organ models will provide an important asset for future approaches to personalized cardiovascular medicine and improved patient care. However, certain technical and biological challenges remain, making the global utilization of OoCs to tackle unanswered questions in cardiovascular science still rather challenging. This review article aims to introduce and summarize published work on hearts- and vessels-on chips but also to provide an outlook and perspective on how these advanced *in vitro* systems can be used to tailor disease models with patient-specific characteristics.

## 1. Introduction

Cardiovascular diseases (CVDs) are a group of disorders affecting the heart and the vasculature that represent the number one cause of mortality globally.^1^ Only in Europe, CVDs causes over 4 million deaths each year,^2^ accounting for 47% of all deaths in Europe. The most common underlying pathology in CVDs is atherosclerosis, which causes an ischaemia in the heart and in peripheral arteries. Atherosclerosis is defined as a chronic disease of the vasculature, whose architecture is slowly remodelled over time. This disease and remodelling process involves the interplay of numerous cell subtypes, including endothelial cells (ECs) (becoming dysfunctional), leukocytes and macrophages (triggering inflammation), and smooth muscle cells (which dedifferentiate or undergo apoptosis).^3^^,^^4^

Unstable atherosclerotic plaques can rupture, which results in arterial thrombosis. Thrombosis, the formation of blood clots, prevents blood flow, and triggers life-threatening clinical conditions in the arterial system, such as myocardial infarction (MI) and ischaemic forms of stroke (IS). Although MI and IS are usually acute events resulting from chronic atherosclerotic processes affecting coronary and carotid arteries, respectively, venous thromboembolism (VTE) is mainly caused by haemostatic or coagulation abnormalities.^5^

Another severe result of tissue ischaemia is heart failure (HF), which is most commonly associated with coronary artery disease (CAD).^6^ Other complications of ischaemia include arrhythmias caused by discontinued oxygen supply to the cardiac conduction system. In particular long-term complications after an acute MI that triggers pathological myocardial remodelling remain unsolved and are a major cause for high re-hospitalization rates.^7^ Hallmark features of this cardiac remodelling process^8^ are excessive deposition of extracellular matrix (ECM) leading to cardiac fibrosis, chamber dilation (dilated cardiomyopathy), and cardiomyocyte hypertrophy. Apart from ischaemia being the key inducer leading to HF, several non-cardiac therapies can cause adverse reactions that induce a similar disease phenotype.^9^ In particular, anti-cancer treatment strategies (radiation as well as chemotherapies) are particularly known for their cardiotoxic potential. Here, the new field of cardio-oncology aims at improving our understanding of molecular and clinical alterations that cancer therapies generate in the cardiovascular system.^10^

Our knowledge about etiopathogenetic mechanisms in CVD has dramatically benefited from advances in ‘-omics’ technologies. A typical pipeline to tackle unanswered disease research questions exploits disciplines such as genomics and transcriptomics to identify novel targets in human cohorts as well as *in vivo* models to validate these findings. Genome-wide association studies (GWAS) to investigate CAD,^11^ VTE,^12^^,^^13^ IS,^14^ and HF^15^ have evaluated hundreds to thousands of individuals and led to the identification of multiple genetic loci associated with the respective disease. With the availability of new technologies and the combination of genomic data utilizing publicly available expression datasets, the translation from genomic loci to the discovery of causal genes is starting to become a reality. Functional assessments using *in vitro* modulation in cultured cells or *in vivo* animal models are considered irreplaceable to identify the actual genes or variants that are relevant for causing the associations while trying to validate biological relevance. However, the translation of discoveries across species remains challenging: the poor sequence conservation of most non-coding genes (unlike protein-coding genes) substantially limits the experimental studies of exiting candidates, such as long non-coding RNAs or circular RNAs.^16^ Moreover, the human circulatory system, including its mechanical, electrical, biochemical, and cellular complexity, is hard to mimic. Human *in vitro* models that consist of single types of cells cultured under static conditions on a plastic surface in two dimensions poorly represent our physiological constitution.^17^ These simplistic models lack the three-dimensional complexity of the tissue, the effect of flow, the cell–cell interaction between blood cells and the endothelium as well as the involvement of the ECM that characterizes vascular tissue *in vivo*.

In recent years, organs-on-chips (OoCs) have emerged as powerful new tools to fill the translational gap from animal models to human disease, with a particular potential to even replace animal testing in the future.^18^ OoC technology will improve the modelling of organs or organ systems for healthcare research while immensely impacting the precision medicine approach.^18^ OoCs comprise systems integrating either 2D cell cultures on permeable membranes or cells cultured in 3D hydrogel scaffolds. In this current review article, we are referring to OoCs as defined by the EU project ORCHID.^19^ Within the scale of novel physiologically relevant *in vitro* models, organoids need to be mentioned at this point. They will however not be covered in greater detail in this present review. Organoids are self-organized three-dimensional tissue cultures deriving from stem cells. They differ from OoCs especially in biological complexity, displaying multi-cellular self-assembled constructs,^20^ whereas OoCs are typically multi-structural engineered systems for on-chip cell cultures.

In the following pages, we introduce the general concept and scientific potential of OoC systems in CVD research. We further discuss the opportunities and challenges of utilizing OoCs in preclinical drug testing and target discovery.

## 2. Methods

**Figure 1:**
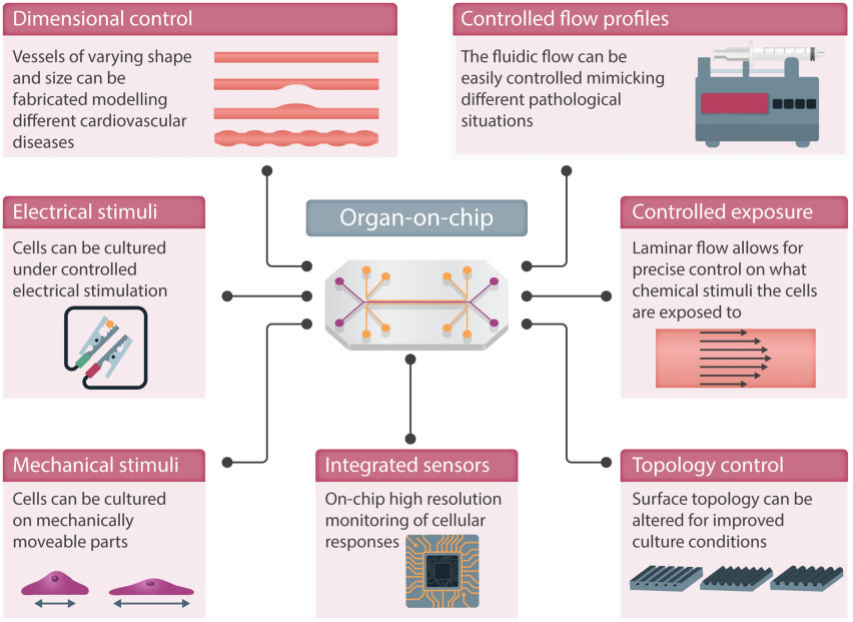
Fabricating heart-on-chip and vessel-on-chip models using micromachining allows for integration of several advanced features.

OoCs are micro-engineered *in vitro* models that recapitulate aspects of human physiology and pathology. They can be used in drug discovery as well as for efficacy and toxicology testing.^21^ OoCs are defined as microfluidic cell-culture devices that contain continuously perfused chambers being inhabited by living human cells arranged in a three-dimensional organization that preserves and mimics the tissue geometry.^22^^,^^23^ The high level of control, enabling customised cell-culture environment in OoCs, is illustrated in *Figure 1*. This includes custom ECM topology, the integration of sensors and actuators for monitoring and electrical/mechanical stimuli, control of microfluidic channel dimensions, and temporal and spatial flow profiles for pulsatile flow and chemical stimuli. Moreover, the precise microfluidic flow control enables an optimal growing environment (influx of nutrients and efflux of cell-waste) as well as the circulation of drugs, signalling molecules, or immune cells. The fabrication methods used to realize OoC systems were originally developed for the microelectronics industry, providing methods to define microfluidic channels and compartments with dimensions from a few micrometres (µm) to several millimetres (mm), thus matching the dimensions of real arteries, veins, and functional units of organs.

**Figure 2:**
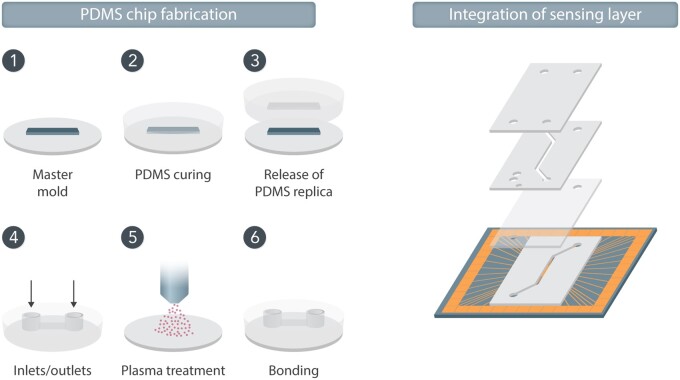
Schematic drawing showing the six basic steps of PDMS moulding to form a microfluidic channel that can be used in organs-on-chip. 1—A master is prepared having the inverse topography of the final channel structures, 2—PDMS pre-polymer is poured onto the master and polymerised upon heat treatment, 3—The moulded channels are released from the master, 4—Holes for connecting tubing for media perfusion are prepared in the PDMS by punching, 5—The PDMS surface is activated for bonding via plasma treatment, 6—The microfluidic channels are sealed by bonding the PMDS slab onto a glass microscope slide which may include patterned electrodes.

In order to fabricate vessel models, it is imperative to consider the geometry as this affects the flow profile, wall shear stress, culture area, and the total number of cells used in the specific model. The material most commonly used for proof-of-concept models is poly(dimethylsiloxane) (PDMS), a polymer developed in the late 1990s ^24^ to fabricate microfluidic channels. PDMS has several advantages for miniaturized cell cultures, such as simple fabrication, gas permeability, and optical transparency. Using PDMS, supportive microfluidic channels can be moulded off a master that has the reverse topographical features. By removing the PDMS from the mould and bonding the structure onto a glass slide, a sealed channel structure can be created (*Figure 2*). Inlet and outlet holes for connecting tubing for perfusion can easily be punched into the PDMS.

In order to form vessel mimics, the microfluidic channels can then be coated with human ECs, forming artificial intima layers of vessels in which blood, plasma, or other cells of interest (e.g. monocytes and platelets) can be perfused.^25^ Specific culture media with different added stimuli or drugs can be added to the system, and real-time observation of the system under a microscope can be used to evaluate the effect of certain stimuli. At the same time, variations in flow and shear stress can provide more information than static cultivation in well plates. To study disease onset, systems with higher complexity, including 3D lumen-chips that incorporate the media layer populated by SMCs, are required. A recent example of a perfusable artery-on-chip was published by Cho and Park,^26^ where SMCs and human umbilical vein ECs (HUVECs) were co-cultured in a PDMS channel. In an attempt to induce the proper morphology and orientation of SMCs, wrinkles were formed on the circular PDMS channel surface during the moulding as contact guidance, and the HUVECs were aligned by medial perfusion.

One major disadvantage of using PDMS is that the material is porous, thus absorbing especially hydrophobic compounds, which can lead to false-negative read-outs from drug-screening studies.^27^ Also, the polymer is not inert, meaning that silicon will leach from the structure into the cell culture environment.^28^ Developments of more inert yet biocompatible materials are therefore currently a major focus within the field.^29^

In an effort to make the vascular structure more biomimetic, one can design the system to include multiple microfluidic compartments where some can be filled with a biomimetic cell-culture scaffold to recreate the physiological environment of the vasculature.^30–33^ Alternatively, one can mould the complete device in a biological material such as collagen.^34^^,^^35^ Cells can further be introduced into the biomimetic scaffolds. This set-up allows investigation of the tissue-blood interface in a controlled environment that cannot be monitored in animals or patients. It can also be utilized to study the interaction with adjacent cells on disease sub-phenotypes, such as endothelial permeability, communication with blood cells, or platelet aggregation. Another approach to fabricate vessel models in a biomimetic material is to utilize the method of viscous fingering,^36^ thus forming co-centred channels instead of adjacent ones. The approach also has the advantage of generating circular vessel structures, which cannot be obtained *via* conventional PDMS moulding. The method has been used to study cell–cell interactions in 600 µm wide^37^ and 250 µm^38^ diameter vessel models. Although very interesting from a biological perspective, there are several technical challenges with integrating biomimetic scaffolds into OoC systems, which have been discussed in more detail elsewhere.^39^

One option to exploit the angiogenic potential of the cells themselves is to increase the biological relevance of the vasculature models even further. In such systems, vascular cells are seeded in the biomimetic scaffold and exposed to mechanical stimuli *via* slow perfusion^40^ or a chemical gradient of growth factors.^41^ This results in a vascular bed formation after 2–3 weeks, including both larger macro-vessels and dense capillary microvascular networks. Taking a completely different approach to investigating small and large artery diseases, one can utilize the advantages of microfluidics by developing advanced *ex vivo* culture platforms. They can be used if a higher complexity is needed to investigate, for example, cell–cell interactions and their relevance for the onset of a certain disease. An initial attempt to investigate structural changes occurring within the vessel wall was presented by Günther *et al.*^42^ They used a microfluidic platform for immobilizing small arteries obtained *ex vivo* from mice and long-term culturing under physiological conditions (37°C, 45 mmHg transmural pressure). Live imaging allowed them to determine the arteries’ inner and outer diameter in real time while assessing the effects of heterogeneous environmental changes on the microvascular structure and function.

Cardiac models are often realized using the multichannel approach described above, as it provides the possibility to compartmentalize the different domains of the cardiovascular system. This unprecedented modularity has led to the development of different heart-on-chip models that have focused on specific cardiovascular subdomains of great interest to cardiovascular researchers. The heart models can be fabricated to include multiple channels that are aligned in parallel either along the horizontal axis ^43–45^ or the vertical axis.^46–48^ The choice of layout is often related to the assays used, where horizontally structured channels, for example, allow for easy optical access of the separate compartments.

There are also several reports of 2D-based cardiac models,^46^^,^^47^ for example developed with an electrophysiological focus due to the increased simplicity of integrating planar electrodes with cell cultures. These systems are often fabricated in more robust materials, such as silicon- or glass-coated, with fibronectin, that enhance cellular adhesion.

Three-dimensional models, on the other hand, are more commonly found in models that mimic multi-organ systems or three-dimensional aspects that 2D models fail to recapitulate, such as the force of contraction measurements and maturation of the co-culture micro-tissues. Such models include mechanically moveable parts, such as suspended membranes^49^^,^^50^ or cantilevers structures^51^ that expose the encapsulated cardiac cells to mechanical stimulation during the culture.

A fabrication method that is rapidly gaining attention, both for realizing vessels and heart constructs, is ‘bioprinting’, a type of additive manufacturing or ‘3D printing’. In bio-printing, hydrogel cell suspensions are directly extruded onto a substrate, building up the final biological structures layer-by-layer. Advantage of bioprinting is that the final device can include a high level of topographical complexity and that multiple cell and material combinations can be realized. Bio-printed models of vascular networks^52^ have been reported, and recently, proof-of-principle of a drug toxicity screening heart-on-chip model using bioprinted cardiac cells was demonstrated.^53^ Although bioprinting is a rapidly developing technology, the challenge with interfacing bioprinted heart models with vascular models for controlled media perfusion still remains.

### 2.1 Integrated electrodes for cellular stimulation and read-out

The inclusion of electrodes in cell-culture systems assist in producing highly controlled environments and allow for continuous read-out of parameters essential to identifying cell behaviour. Various materials can be used for the fabrication of the electrodes, including bio-friendly metals such as gold^44^ and platinum,^46^^,^^54^ and organic conducting materials, such as carbon^55^ or poly (3,4-ethylenedioxythiophene) polystyrene sulfonate (PEDOT: PSS).^56^

For the realization of reliable heart-on-chip models, it is very important to support the development of cells with a mature electrophysiological conduction system, such as synchronized beating. Although some cultures of cardiomyocytes show spontaneous synchronized beating already after a few days of culture even without external interactions,^44^^,^^54^^,^^57^ an effective method to induce synchronised beating in immature cultures is to assist the synchronization of the beating by using external electrical stimulation^58^^,^^59^ (*Figure 2*). Typically, the cells are exposed to trains of electrical pulses similar to the electrical signalling of native cardiomyocytes. Often, this is achieved *via* large external electrodes, but more user-friendly custom systems have been developed using integrated electrodes on-chip.^44^^,^^60^ It has been shown that maturation of cells may be improved by pacing the cells as proved by expression of α-actinin, connexin,^43^ and cardiac troponin-T.^60^ In addition to external electrical stimulation, maturation schemes that rely on external mechanical^43^^,^^61^ and biochemical^62^ cues—or a combination of the aforementioned^50^^,^^57^—have also been presented.

The main use for microfabricated integrated electrodes in heart-on-chip systems is, however, for on-chip read-out of electrophysiology. Here, the ion currents of the cells are measured extracellularly as changes in the field potential. The extracellular field potential is closely linked to the QT interval through the corrected field potential duration.^63^ Multiple localized measurements of single cells enable assessment of the synchronization of beating cells and wavefront propagation. As demonstrated in open-format cell cultures, cells can be cultured on top of high-density electrodes, so-called microelectrode arrays (MEAs) for read-out of cellular electrophysiology.^64–66^ Customized MEAs can fit in virtually any microfluidic system and have been reported for several OoC systems.^46^^,^^54^^,^^67^

Normally, very close contact between the cells and the MEA is desired for optimal resolution and signal strength, resulting in cell culture on hard flat surfaces and 2D cell models. To make the culture environment more biomimetic, Kujala *et al.*^64^ cultured cardiomyocytes on a ∼100-µm-thick micro-grooved gelatin layer attached to the MEA and showed that the electrophysiology still could be mapped, although no longer with single-cell resolution. Other approaches that address the issue of 2D culture on hard surfaces are non-contact MEA measurements as explored by Sharf *et al.*^68^ and patterning of MEAs on a soft PDMS substrate as demonstrated by Gaio *et al.*^57^

Electrical sensors can also be integrated to monitor cell contraction. Quin *et al.*^65^ have demonstrated this in an open-top structure, where interdigitated electrodes were pattered for cell-contraction measurements in combination with MEAs to monitor beating. Although highly correlated in normally functioning cardiac tissue, contraction and electrophysiology are two different mechanisms that are important to follow in heart-on-chip models. Alternative ways to map cell contraction is to integrate mechanical sensors into the heart model,^54^^,^^66^ or to utilize an external optical read-out with computational motion tracking,^62^ or mapping the transient intracellular calcium signal using cells modified to include GCaMP6.^44^

Integrated electrodes are also interesting for vessel models, as they can be used to assess the barrier integrity of the cultured cells *via* trans-endothelial electrical resistance (TEER) measurements. Most commonly, this method works by integrating electrodes on either side of a porous membrane on which the ECs are cultured. As the cells form tight junctions, it becomes increasingly more difficult for any electrical current to flow through the cell layer, and the measured electrical resistance increases. It is possible to combine electrophysiological measurements and TEER as demonstrated by Maoz *et al.*,^46^ using a dual-channel, endothelialized heart-on-chip model. Further, 3D tubular vessels may incorporate TEER sensing capability by insertion of electrodes inside the vessel and in a surrounding hydrogel matrix.^69^

It may be noted that most models with integrated electrical sensing capabilities are two-dimensional, which is explained by the well-established technology to form electrodes on hard 2D surfaces. However, electrodes can also interface more *in vivo*-like cardiac microtissue as in the case of Weng *et al.*^44^ An increase in the number of publications on this topic is expected. Further, the hydrogel scaffold may be topologically patterned on top of the electrodes,^64^ or the electrodes themselves can be structured into 3D formats.^60^^,^^70^ Alternatively, conducting and biocompatible scaffolds can be prepared, thus enabling electrical read-out *via* the porous scaffold itself.^71^

### 2.2 Heart-on-chip disease models

**Figure 3:**
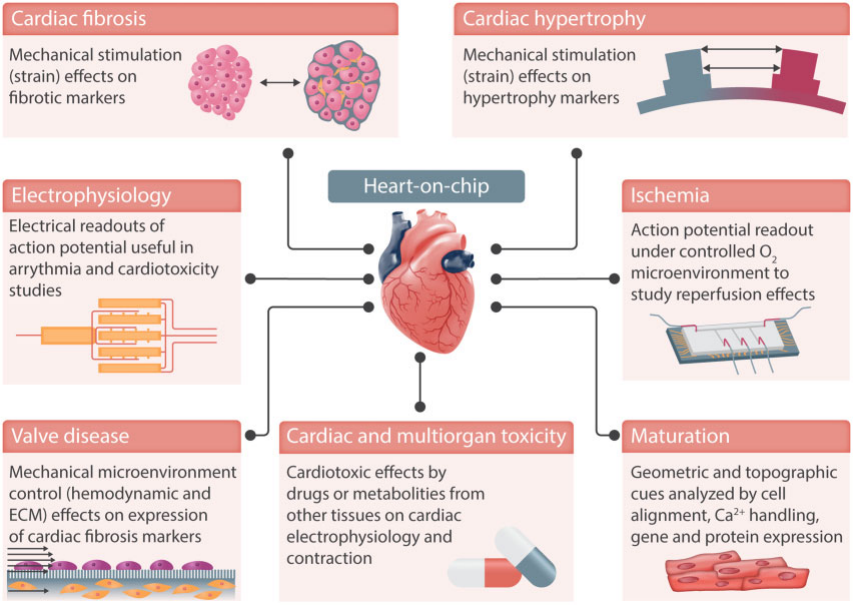
Heart-on-chip devices can recapitulate cardiac functions *in vitro* and integrate sensing units to monitor the cells in culture, e.g. action potential. Examples of cardiovascular diseases can be found in these devices, such as ischaemia and cardiac fibrosis. Integrated electrodes and mechanical actuation allow to monitor and stimulate the cells in culture, better recapitulating the cardiac microenvironment.

Heart-on-a-chip models developed so far have focused on establishing biomimetic, functional aspects of the heart, focusing in particular on the co-culture of multiple cell types, for example, cardiomyocytes and cardiac fibroblasts as well as on the electromechanical stimulation of the cells cultured on these systems (*Figure 3*). Heart-on-chip models aim to recapitulate the intricate conditions of the microenvironment that cells would experience in the heart. Nonetheless, the ability to include 3D cell cultures in these devices enables an increase in their biological complexity. Control over the microenvironment and the cultured cell types permits the recreation of relevant aspects of a specific disease (*Table 1*). Among the different reported heart-on-a-chip platforms, models to address cardiac ischaemia, cardiac fibrosis, and cardiotoxicity can be found in the literature (*Figure 3*), whereas many other exiting approaches have been developed and summarized in further review articles.^62^^,^^72–77^

**Table 1:** Summary of the heart-on-chip platforms.

**Aspects of human cardiac physiology and disease in organs-on-chips**		
Cardiac physiology	Defined 3D tissue organization	^43^ ^,^ ^48^ ^,^ ^61^ ^,^ ^62^ ^,^ ^64^ ^,^ ^135^
	Force of contraction	^51^ ^,^ ^54^ ^,^ ^130^ ^,^ ^135^
	Electrophysiology	^43^ ^,^ ^46^ ^,^ ^57^ ^,^ ^62^ ^,^ ^64^ ^,^ ^65^ ^,^ ^67^ ^,^ ^135^
	Cardiac-vascular interactions	^44^ ^,^ ^46^ ^,^ ^62^ ^,^ ^135^
	Body-on-chip approach	^54^ ^,^ ^130^ ^,^ ^135^
Cardiac disease and toxicity	Hypertrophy	^61^
	Arrhythmia	^47^ ^,^ ^49^
	Ischaemia	^67^
	Fibrosis (e.g. fibroblast proliferation, collagen deposition, and valve calcification)	^47^ ^,^ ^49^
	Inflammation	^46^ ^,^ ^135^
	Cardiotoxicity & Pharmacology	^43^ ^,^ ^44^ ^,^ ^46^ ^,^ ^51^ ^,^ ^54^ ^,^ ^61^ ^,^ ^62^ ^,^ ^64^ ^,^ ^65^ ^,^ ^130^ ^,^ ^135^

The selection criteria employed in this table were that the devices used in the study could be considered a heart-on-a-chip device, i.e. a microfluidic device where the microenvironment of the cells or tissue in culture can be controlled and/or be stimulated mechanically and/or electrically. Platforms where constructs are cultured in well-plates or make use of spheroid technology were not considered due to the lack of their microfluidic character, which is seen as a requirement for organs-on-chips.

#### 2.2.1 Ischaemia

The abrupt disruption of blood flow in ischaemia leads to the accumulation of metabolic by-products while reducing the oxygen supply to the tissue. This locally affects the contraction of cardiomyocytes. Liu *et al.*^67^ were able to replicate the hypoxic microenvironment and follow the action potential changes over time in the cell culture using patterned electrodes in a heart-on-a-chip device. The combination of the microenvironment cues, along with the insights gained from the electrophysiology of the cells depicts the solid control that can be attained with these devices.

#### 2.2.2 Cardiac fibrosis

In the scope of cardiac fibrosis, Kong *et al.*^49^ were able to recreate the increased ECM stiffness using a photopolymerizable hydrogel while including the mechanical load similar to the stimulus that cardiac fibroblasts would experience under pro-fibrotic conditions. The cyclic mechanical loading, along with the exposure to a biochemical stimulus like transforming growth factor β (TGFβ) can even more closely mimic the fibrotic microenvironment of the heart. There have also been non-microfluidic devices that make use of 3D cardiac tissue models to mimic hallmarks of cardiac fibrosis using human induced pluripotent stem cell-derived cardiomyocytes (iPSC-CMs) along with fibroblasts. Mastikhina *et al.*^78^ reported a model where expression of collagen and brain natriuretic protein were upregulated when tissues were exposed to TGF-β, a pro-fibrotic agent, and downregulated when subsequently exposed to an anti-fibrotic drug. Wang *et al.*^35^ used cardiac fibroblast overpopulation of the tissues to mimic a fibrotic scenario, thus avoiding the pleiotropic effects of TGF-β. These two methods could be combined into microfluidic devices in additional adjacent compartments where, ECs can be integrated, and more complex, integrative models be made.

#### 2.2.3 Cardiotoxicity

Interestingly, the vast majority of heart-on-chip devices reported in literature state cardiotoxicity as their primary goal. Although cardiotoxicity encompasses a myriad of aetiologies, two common trends can be found in these devices: (i) an investigation of the toxic effects that specific drugs have on cardiac cells and (ii) the toxic effect produced by the co-cultured cells from other organs (e.g. liver). The highly controlled environment of the heart-on-chip devices makes it a highly appealing platform for drug toxicity studies, as evidenced by a large number of devices with this as its motivation of the design.

The highly controlled microenvironment in heart-on-chip models makes them very useful for modelling diseases. There is plenty of room to study disease mechanisms that cannot be dissected in common platforms such as cell culture and animal models. However, one advantage of animal models compared with heart-on-a-chip models is the cross-talk between different organs, which are typically involved in the chronic diseases that lead to CVD.^79^ A major challenge in developing heart-on-a-chip disease models would be the difficulty of integrating multi-organ co-cultures for longer periods of time, typically around 1 week–7 days—after tissue formation. Nonetheless, aspects of complex chronic diseases can still be modelled with OoCs, e.g., cardiac fibrosis, as mentioned above.

Naturally, the aforementioned models make use of cardiac cells, a scarce resource since primary cardiomyocytes proliferate poorly *in vitro.* With the advent of human iPSC-CMs, this major cell source bottleneck is gradually being overcome. However, hurdles still remain to improve their immaturity, which ultimately affects the pharmacological response, with oncotherapy effects^80^ and the biomimetic aspects of the OoCs that employ human iPSC-CMs. Cardiac cell maturity can be defined in several aspects such as electrophysiology, metabolism, and morphology, to name a few. Unveiling what mechanisms underlie iPSC-derived cardiomyocytes maturity is an active field of research and is reviewed elsewhere.^81^ The models created with these cells naturally inherit their limitations. There are examples of initiatives that aim to characterize iPSC-CMs and their response to pharmacological agents with a known response as an attempt to benchmark different cell sources, such as the CiPA initiative^82^ and the Pulse CRACK-IT^83^ project. In the future development of heart-on-chip models, the drugs used for benchmarking in the mentioned initiatives can be used as a reference for new devices. With the aim of generating a model where the response of cardiac tissues exposed to libraries of compounds could determine the drug used, Lee *et al.*^84^ used a machine-learning model for the drug-response analysis. This clearly demonstrates that heart-on-chip development is a multidisciplinary field, where pharmacologists, engineers, biologists, regulators, and clinicians, among others, play a key role in the development of these models and their respective validation.

### 2.3 Studying atherosclerosis using chip-based systems

**Figure 4:**
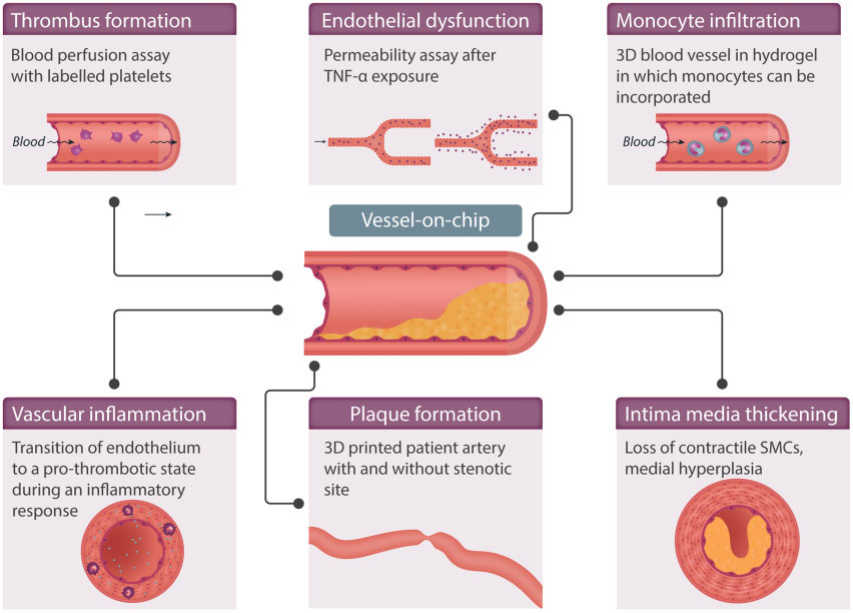
Vessels-on-chips devices are useful tools to study pathological mechanisms occurring within the vessel wall in early and later stages of atherosclerosis. Preliminary research can be performed in easier fabricated straight PDMS channels, whereas for more extensive and complicated research questions, a more elaborate model can be used by producing a 3D lumen in a hydrogel.

Modelling the different stages of the advancement of atherosclerosis is considered crucial in the development of vascular OoCs (summarized in *Table 2* and *Figure 4*). Qiu *et al*.^25^ developed a 3D microvasculature-on-chip that is able to display physiological endothelial barrier function for several weeks, therefore allowing to study how chronic endothelial dysfunction develops. To model atherosclerosis progression, several devices have been created with occlusion or stenosis of the lumen, which recreates the higher shear region commonly found in developing atherosclerotic plaques.^38^

**Table 2:** Summary of vessel-on-chip platforms.

Aspects of human vascular physiology and disease in organs-on-chips		
Vascular physiology	Perfusable 3D blood vessels with defined geometries	^37^ ^,^ ^38^ ^,^ ^87^ ^,^ ^93^ ^,^ ^97^ ^,^ ^139^ ^,^ ^140^
	Perfusable microvasculature with self-organized geometries	^31^ ^,^ ^141^
	Angiogenesis	^31^ ^,^ ^34^
	Endothelial-mural interactions	^31^ ^,^ ^34^ ^,^ ^37^ ^,^ ^139^
	Blood perfusion	^34^ ^,^ ^85^ ^,^ ^87^ ^,^ ^94^ ^,^ ^98^ ^,^ ^99^
Vascular disease modelling	Thrombosis	^34^ ^,^ ^85^ ^,^ ^87^ ^,^ ^93^ ^,^ ^94^ ^,^ ^97–99^
	Inflammation (e.g. permeability and adhesion molecules)	^31^ ^,^ ^34^ ^,^ ^37^ ^,^ ^85^ ^,^ ^93^ ^,^ ^94^ ^,^ ^97^ ^,^ ^98^ ^,^ ^139^
	Immune cell recruitment	^31^ ^,^ ^34^

Vessel-on-chip devices included in this table consist of different types of perfusable blood vessels modelling human vascular physiology as well as pathophysiology.

Westein *et al*.,^85^ Tovar-Lopez *et al*.,^86^ and Costa *et al*.^87^ have provided excellent examples of how to investigate atherosclerosis using chip-based systems. These three models differ regarding their biological and technical complexity. The chips developed by Westein *et al.* and Tovar-Lopez *et al.* consist of a square channel in which an artificial atherosclerotic plaque is already embedded (*Figure 4*). In order to study 3D vessel geometry in a more physiologic way, Costa *et al.*^87^ created different 3D vascular structures by using 3D-printed anatomical models based on observations generated by computed tomography angiography. They were, therefore, able to closely mimic architectures found in both healthy and stenotic blood vessels.

A disadvantage of these models is the lack of the ECM, as PDMS is responsible for creating the lumen that directly surrounds the cells. If the occlusion mechanism and pathophysiology of the development of atherosclerosis are to be studied, a more flexible model in which monocytes and macrophages can be included would be highly appreciated.

### 2.4 Mimicking thrombosis on-a-chip

Thrombosis is a complex process influenced by genetic and environmental factors, involving three main components: abnormalities in the vessel wall (endothelium), abnormalities in components of the blood (coagulation proteins and platelets), or abnormalities in fluid dynamics (turbulence flow and shear stress).^88^^,^^89^ Unlike static *in vitro* cell-culture models, vessels-on-a-chip can mimic the effect of flow and its interaction with the vessel wall while overcoming inter-species differences of animal models. Microfluidic technology has therefore been used extensively to create new *in vitro* models to study thrombotic diseases.^90–94^

Although previous work has been published studying thrombosis on microfluidic devices lacking ECs (reviewed in Westein et al.^91^ and Zhu et al.^92^), we here review OoC models of thrombosis typically consisting of a tubular or rectangular mould that is covered by components of the ECM (typically fibrin or collagen). ECs form a monolayer, therefore recreating endothelial geometry and function. In normal healthy conditions, the endothelium has anticoagulant and anti-inflammatory properties, therefore allowing blood flow through the lumen and preventing platelet activation and fibrin clot formation.^95^ Upon endothelial damage or activation, coagulation and platelet aggregation are triggered to promote the formation of a thrombus.^96^ Besides endothelial damage, several stimuli and the effect of blood flow are known to activate the endothelium.

Several examples of OoC have been published that study the different triggers of clot formation through direct monitoring of platelet aggregation under a microscope. In this direction, Zheng *et al.*^34^ engineered microvascular networks by seeding HUVECs intro microfluidic circuits coated with collagen and demonstrated that upon perfusion with human blood, rolling and adhesion of platelets occurred only at sites of damage—or in stimulated endothelium where long fibres of von Willebrand factor (VWF) covered the surface of the activated endothelium. These structures had not been reported in previous planar cultures or mouse models of thrombosis. Similarly, a recent study by Brouns *et al*.^94^ explored the anticoagulant effect of intact endothelium (created by a microfluidic model coated with HUVECs) through natural anticoagulants while comparing with artificially damaged endothelium covering a highly thrombogenic surface created with collagen and tissue factor.

One of the key advantages of OoCs is their great capacity to tightly control vessel geometry and flow, which has contributed significantly to the understanding of flow-based changes on platelet activation and the risk of thrombosis. To better understand the contribution of atherosclerosis and vessel stenosis to thrombosis, stenotic vessels perfused with whole human blood have been developed. The results indicate that platelets aggregate at the outlet zone of constriction, therefore concluding that the shear rate is crucial for platelet adhesion and aggregation.^85^^,^^87^^,^^90^^,^^97^

Although these examples clearly contribute to advancing research and understanding of the coagulation process, most of these models are not practical to enhance clinical diagnosis. Several commercial devices are emerging with the idea to provide simple tools that can be used for clinical testing. Towards this aim, Mannino *et al.*^97^ proposed a commercial simple endothelial-coated cylindrical microchannel to test the effect of local vascular geometries on blood cell-endothelium interactions. One year later, Jain *et al.*^98^ demonstrated that a microfluidic device coated with ECs could be fixed and still retain their ability to modulate haemostasis under flow. The device was able to detect differences in patients taking antiplatelet medication and therefore providing a new tool for clinical laboratories for coagulation testing in more robust and practical diagnostic assays.

Mathur *et al*.^99^ created a model for thrombo-inflammation where they first isolated blood outgrowth endothelial cells (BOEC) from healthy subjects and then introduced these cells to microfluidic channels. These cells were then stimulated with TNFα and exposed to re-calcified human whole blood to compare the inflammatory response of these cells to models using HUVECs. Due to the easy isolation method of these BOECs, and a different response of healthy and unhealthy patients, this model could be used as a tool in personalized medicine approaches for certain pathologies, in which thrombo-inflammation is an essential contributor to disease exacerbation.

Sepsis is a disease often coinciding with thrombus formation in smaller vessels and arteries, which can lead to ischaemic events of the heart and subsequent HF.^100^ Many of the models described above can be used to model thrombotic sepsis in a microfluidic chip. In sepsis, high levels of inflammation in blood vessels are observed and accompanied by an elevation of TNF-α, interleukin-1, and interferons. These factors activate the endothelium to a pro-thrombotic state and strongly induce thrombus formation.^101^^,^^102^ After incubation with these factors, or other known contributors to sepsis, a blood perfusion assay can be performed to better understand more mechanisms involved in sepsis-associated thrombosis.

Finally, clinical applicability has also been demonstrated by using vessels-on-chips to predict thrombotic side effects in drug candidates prior to human clinical trials. For example, Tsai and collaborators used a vessel-on-chip device to perfuse blood samples from patients with sickle cell disease and test the effect of certain drugs in microvascular occlusion and thrombosis.^93^ Another study by Barrile *et al.*^103^ used a vascular channel coated with ECs and perfused whole human blood to study the potential for different drugs to promote blood clots. These studies serve as interesting examples for the potential of vessels-on-chips to evaluate thrombotic side effects that would otherwise be missed in prior animal or static cell culture studies. They are further proof of how OoCs can enhance drug safety during the process of developing novel treatment strategies.

### 2.5 Future importance of organs-on-a-chip in personalized cardiovascular medicine

Animal and genetic studies have implicated specific genes conferring increased susceptibility to many human diseases. Together with other emerging techniques (e.g. genome editing), OoCs have the potential to significantly enhance our understanding of how certain genes and epigenetic regulators are capable of influencing our personal CVD risk.

The novel genome editing CRISPR/Cas9 technology enables the introduction of targeted mutations or specific gene knock-outs^104^ into human cells. This methodology has allowed the generation of stable cell lines carrying the desired genetic mutation/knock-out while eliminating the effect of inter-individual variations due to the genetic background.

Although primary cells from specific individuals can be difficult to obtain and cannot be cultured indefinitely, stable cell lines with the desired genomic background can be generated from human iPSCs. Human iPSCs are obtained from a somatic cell and can be differentiated into all cell types.^110^^,^^111^ They can therefore be derived from accessible adult tissues of any patient, such as the blood or the skin. There are current protocols available for the generation of cardiomyocytes,^112^ defined atrial and ventricular cardiomyocyte subtypes,^113^ ECs as well as pericytes and vascular SMCs.^114^^,^^115^ An important limitation of iPSC-CMs relates to their immaturity. In fact, they share more similarities with foetal than adult human CMs. Although human iPSC-CMs express high levels of cardiac-specific genes and display a striated pattern for α-actinin and myosin light chain similar to the adult ventricular myocardium, their shape is typically roundish (and not elongated), whereas their cell body is smaller.^111^ In addition, the intrinsic immaturity of iPSC-CMs is reflected by electrophysiological impairments,^111^ which constitute a valid criticism when employing these cells for the investigation of arrhythmias. Various methods to enhance maturity are constantly developed and refined, including exposure to electrical stimulation, application of mechanical strain, and culturing human iPSC-CMs in three-dimensional tissue configuration.^112^ A great advantage of the iPSC differentiation protocols is that they allow for patient-specific iPSC-derived systems that can be conveniently edited to test the effect of specific disease-related mutations.^113^^,^^114^ The aforementioned gene-editing can be used to restore the effect of specific genes or mutations while exploring genotypic effects on specific phenotypes.

The opportunity to build OoC with disease-relevant cells can minimize the current challenges of most genomic studies. For example, vessel- and heart-on-chip designs could be used to test whether genetic associations emerging from recent CAD, VTE, HF, or IS GWAS will increase the disease risk through an effect in the cardiovascular system. At the same time, blood from individuals with a known genetic background can be perfused into different microchannels to study interactions between blood cells and the endothelium. Overall, the advanced possibilities of OoCs using novel available technologies offer great potential to finally elucidate the effect of genetic factors on CVD phenotypes, therefore enabling us to more rapidly and efficiently move towards the era of personalized medicine and pharmacogenomics. OoCs have already demonstrated their potential to support clinical trials of certain drug candidates.^120^ The opportunity to create patient-specific organ-on-chips could therefore pave the way for the development of precise and individualized therapies.

It is essential to mention that the majority of advances in personalized medicine stem from organoids-based research. In this context, Atchinson *et al*.^116^ have developed micro-engineered blood vessels based on SMCs derived from human iPSC of Hutchinson–Gilford progeria, a rare accelerating aging disorder that causes early onset of atherosclerosis. The work highlighted the possibility of the *in vitro* system to recapitulate the key features of the disease and how to utilize it as a patient-specific drug testing device. More recently, self-assembled 3D blood vessel organoids have been generated from human iPSCs. Blood vessel organoids are made of endothelial networks and pericytes that are genetically identical and can recapitulate the formation of a vascular lumen and basement membrane deposition.^117^ However, it is not yet possible to mimic blood flow perfusion in these models and therefore to obtain functional parameters, such as permeability or immune-cell adhesion/extravasation under defined *in vitro* conditions. This, as aforementioned, is one of the major advantages of OoC systems.

Moreover, several studies showing the integration of gene-editing technology and organoids culture systems have been published.^118^^,^^119^ An example was presented in a cystic fibrosis organoid model, where mutations in *CFTR* gene were repaired in intestinal stem cells by CRISPR/Cas9 system.^120^ Genome editing in iPSC-derived organoids have also been proved useful for drug testing,^121^ or to study the effect of virus infections.^122^^,^^123^ Very recent work in organoids made from iPSCs have shown that SARS-CoV-2 can infect engineered human blood vessel organoids and leak out into the bloodstream^124^ and that the infection could be inhibited by human recombinant soluble angiotensin-converting enzyme 2 (ACE2).

Few examples of personalized medicine accompanied by OoCs have been published in the CVD field.^125^^,^^126^ Wang *et al.*^127^ created a heart-on-a-chip using genetically engineered iPSC to proof that contractile deficiencies in cardiomyocytes associated with Barth syndrome were caused by a mutation in tafazzin gene, thus elucidating the biological mechanism and providing potential therapeutic targets to treat the disease. Another interesting approach is the integration of clinical data with the fabrication of OoC devices was used by Costa *et al.*,^87^ as described in detail in the atherosclerosis section above. Here, CTA data of a coronary artery formed the basis to construct personalized chips with the measured grade of stenosis. This method allowed the researchers to reproduce *in vitro* the unique (personalized) flow profile of individual patients and to observe the formation of thrombi dynamically. Similarly, blood vessel-on-chips can be perfused with human blood from individuals treated with different anticoagulant therapies to assess drug response of specific patients in a personalized manner.^128^

## 3. Conclusion and outlook

OoCs have advanced the drug development process by stimulating scientists to increase the level of complexity of better mimicking human biology and physiology on a more systemic level. Further, the possibility to integrate more than one organ in the same model is an important and on-going effort in the field.^129–131^ Microfluidic devices that contain the function of different organs have been connected with each other *via* vessels-on-chips to obtain the so-called ‘body-on-a-chip’. This concept captures the potential efficacy and toxicity of a drug in different organs.^129^^,^^132^ An important advancement in the validation of multi-organs-on-chip usage in drug screening has been the integration of sensors to ensure continuous assessment of the microenvironment parameters (pH, oxygen, and temperature).^133^ Kamei *et al.*^134^ have developed an integrated system composed by a healthy heart-on-chip connected to a liver cancer-on-chip and recapitulated the cardiotoxic effect of the anti-cancer drug doxorubicin. The side effect of the doxorubicin treatment on cardiac cells was due to toxic metabolites produced in the liver cancer cells.

Especially relevant for cardiovascular research are models that include both vasculature and the heart. This can be achieved either by building a microfabricated vascular network that cardiac tissue can be shaped around^135^ or by generating vascularized cardiac microtissues *via* co-culture with ECs. The latter approach shows a higher biological relevance but comes with the challenge to interface the microtissue constructs with microfluidic circuits for controlled perfusion. To recapitulate complex, multi-layered, and interconnected tissue architectures remain impossible with the current engineering approaches utilised to build OoCs. However, the possibility to combine biological self-assembly capabilities of organoids with the controllable assembly of microfabricated OoCs represents an attractive advancement of both technologies. This has been demonstrated by a novel microphysiological model of the human retina derived from hiPSCs incorporated in a two-channel chip separated by a thin porous membrane mimicking the endothelial barrier and enabling the exchange of nutrients and metabolites.^136^

An important challenge for cardiovascular OoC platforms is the ability to reproduce the chronic aspect underlying the progression of CVDs. Animal models in this respect are still somewhat irreplaceable; however, investigators can benefit from OoCs to study a specific mechanism and subsequently refine the targets to be evaluated in further animal models.

The field of OoC is growing, and an increasing number of academic groups and companies are becoming active in the development of OoC systems.^19^ All these OoC systems have different layouts and interfaces, which prevents them from being easily connected, interchanged, or compared. This lack of common standards for OoC systems is considered to be one of the major challenges in their future development and implementation.^137^ Initiatives are currently emerging in the field to address this challenge, for example, the development of a Translational Organ-on-Chip Platform, which aims to be an ‘open’ platform based on standards that are defined and supported by the stakeholders (developers, manufacturers, users) in the field (https://top.hdmt.technology/).

Standardization and harmonization are not only of importance for the technical aspects of an OoC but also for the cells and tissues that are integrated into the chips. Human cells are highly variable in terms of growth, stability, and function, particularly when primary tissues or stem cells are used as a source. Mainly, users from the industry consider access to human cell material, including patient material, to be a major challenge.^138^ In the field of stem-cell technology, efforts are made to address this issue by setting up open databases of available human stem-cell lines, including their characteristics, sources, and restrictions to use (e.g. https://hpscreg.eu/).

Overall, the use of OoC is rapidly moving from basic science to translational research to validate results from genomic studies and provide better models for drug testing, paving the way for personalized medicine. The combination of organ-on-chips with gene editing and iPSC use for better control of genetic background is becoming an innovative, attractive alternative for the functional study of the cardiovascular system.
